# Supplementary material for: The transcript cleavage factor paralogue TFS4 is a potent RNA polymerase inhibitor
Source: Nat Commun. 2017 Dec 4;8:1914. doi: 10.1038/s41467-017-02081-3 (PMC5715097; doi:10.1038/s41467-017-02081-3)
Supplement: Supplementary file 1 — Supplementary Information [file 41467_2017_2081_MOESM1_ESM.pdf]

# Supplementary materials and methods

## Supplementary methods

### Potassium permanganate foot-printing

The potassium permanganate foot-printing assays were carried as previously described[1]. 1 μM TBP and 125 nM TFB, 70 nM of RNAP were incubated in transcription buffer containing 26 mM $MgCl_2$ and 0.5 mM DTT, in the presence or absence of 3 μM SsoTFEα/β, 700 nM TFS1 or TFS4. Samples were incubated for 5 min at 65°C before the addition of 4 mM $KMnO_4$. After additional 5 min incubation at 65°C, the reaction was stopped by addition of 1.5 μl 2-mercaptoethanol. After Proteinase K treatment for 1 hour at 50°C, the samples were ethanol-precipitated. Samples were resuspended in 50 μl 1 M piperidine and incubated at 90°C for 30 min. After chloroform-extraction, samples were ethanol-precipitated and resuspended in 12 μl formamide loading dye. 5 μl of the samples were separated on 10% polyacrylamide, 7M Urea, 1×TBE sequencing gel. Gels were dried for 1 h at 80°C under vacuum, visualized using Typhoon FLA 9500 biomolecular imager (GE Healthcare) and analyzed using ImageQuant TL Software (GE Healthcare).

## Supplementary tables and figures

| Super-phylum | Phylum | Order | Representative specie | Number of TFS factor | Accession numbers |
|---|---|---|---|---|---|
| ASGARD | Odinarchaeota | | Candidatus *Odinarchaeota* archaeon LCB_4 | 1 | OLS17806.1 |
| | Lokiarchaeota | | *Lokiarchaeum* sp. GC14_75 | 2 | KKK43059.1; KKK43557.1 |
| | Thorarchaeota | | Candidatus *Thorarchaeota* archaeon SMTZ1-45 | 2 | KXH72617.1; KXH71193.1 |
| | Heimdallarchaeota | | Candidatus *Heimdallarchaeota* archaeon AB_125 | 2 | OLS32995.1; OLS32960.1 |
| TACK | Thaumarchaeota | | *Thaumarchaeota* archaeon CSP1-1 | 1 | KRT60970.1 |
| | Aigarchaeota | | *Caldiarchaeum subterraneum* | 1 | BAJ47392.1 |
| | Crenarchaota | Sulfolobales | *Sulfolobus solfataricus* P2 | 4 | AAK40629.1; AAK40916.1; AAK40629.1; AAK42105.1 |
| | | Acidilobales | *Acidilobus saccharovorans* 345-15 | 2 | ADL18862.1; ADL19509.1 |
| | | Desulfococcales | *Ignicoccus islandicus* DSM 13165 | 2 | ALU11731.1; ALU11838.1 |
| | | Thermoproteales | *Thermoproteus tenax* Kra 1 | 2 | CCC81245.1; CCC81368.1 |
| | Korarchaeota | | Candidatus *Korarchaeum cryptofilum* OPF8 | 1 | ACB07793.1 |
| | Bathyarchaeota | | Candidatus *Bathyarchaeota archaeon* RBG_16_48_13 | 1 | OGD46939.1 |
| | Euryarchaeota | Archaeoglobi | *Archaeoglobus sulfaticallidus* PM70-1 | 1 | AGK60260.1 |
| | | Halobacteria | *Haloferax volcanii* DS2 | 4 | ADE03944.1; ADE05146.1; ADE01865.1; ADE01888.1 |
| | | Methanobacteria | *Methanosphaera stadtmanae* DSM 3091 | 2 | ABC57900.1; ABC57619.1 |
| | | Methanococci | *Methanocaldococcus jannaschii* DSM 2661 | 1 | AAB99148.1 |
| | | Methanomicrobia | *Methanosarcina mazei* Go1 | 1 | AAM31094.1 |
| | | Methanopyri | *Methanopyrus kandleri* | 0 | |
| | | Thermococci | *Pyrococcus furiosus* DSM 3638 | 1 | AAL81110.1 |
| | | Thermoplasmata | *Thermoplasma acidophilum* | 1 | CAC12135.1 |
| DPANN | Diapherotrites | | Candidatus *Iainarchaeum andersonii* SCGC AAA011-E11 | ? | |
| | Parvarchaeota | | Candidatus *Parvarchaeum acidiphilum* ARMAN-4 | 1 | EEZ93161.1 |
| | Aenigmarchaeota | | Candidatus *Aenigmarchaeota archaeon* CG1_02_38_14 | ? | |
| | Nanohaloarchaea | | *Nanohaloarchaea archaeon* SG9 | 1 | AOV94756.1 |
| | Nanoarchaeota | | *Nanoarchaeum equitans* Kin4-M | 1 | AAR39225.1 |

**Table 1: Overview of distribution of TFS paralogues in archaea.**

Distribution of archaeal TFS homologs and paralogs. Members of proposed superphyla based on references [2-4]. Plasmid-encoded factors are underlined.

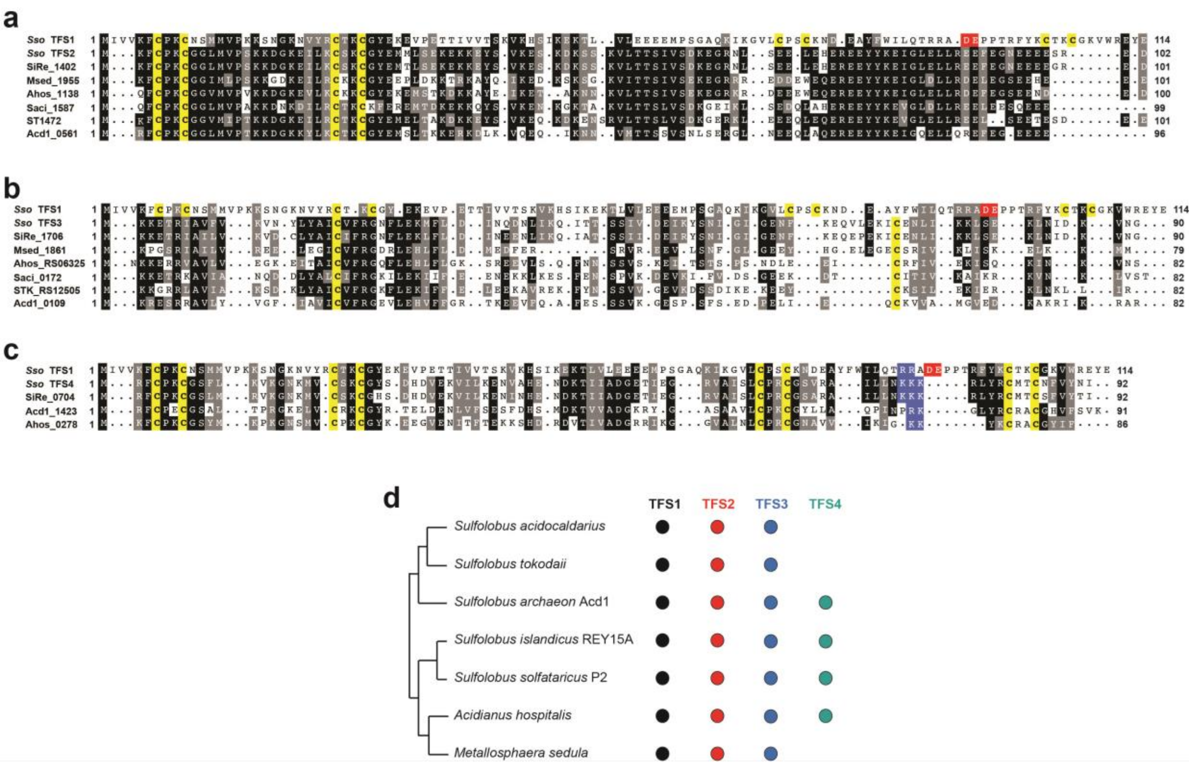


**Supplementary figure 1: Evolutionary conservation of archaeal transcript cleavage factors paralogues.**

**(a)** Amino acid sequence alignment of TFS2, **(b)** TFS3 and **(c)** TFS4 homologues in the Sulfolobales order. (a) Sso TFS2 (AAK40916.1), *Sulfolobus islandicus* REY15/4 SiRe_1402 (ADX85468.1), *Metallosphaera sedula* DSM 5348 Msed_1955 (ABP96095.1), *Acidianus hospitalis* Ahos_1138 (AEE94022.1), *Sulfolobus acidocaldarius* DSM 639 Saci_1587 (AAY80900.1), *Sulfolobus tokodaii str. 7* ST1472 (WP_010979520.1), *Sulfolobus* Acd1 Acd1_0561. (b) Sso TFS3 (AAK40630.1), *Sulfolobus islandicus* REY15/4 SiRe_1706 (ADX85770.1), *Metallosphaera sedula* DSM 5348 Msed_1861 (ABP96001.1), *Acidianus hospitalis* Ahos_RS06325 (WP_048054635.1), *Sulfolobus acidocaldarius* DSM 639 Saci_0172 (AAY79591.1), *Sulfolobus tokodaii str. 7* STK_RS12505 (WP_052846758.1), *Sulfolobus* Acd1 Acd1_0109. (c) Sso TFS4 (AAK42105.1), *Sulfolobus islandicus* REY15/4 SiRe_0704 (ADX84786.1), *Sulfolobus* Acd1 Acd1_1423 and *Acidianus hospitalis* Ahos_0278 (AEE93169.1). The basic residues in the TFS4 C-ZR are highlighted in blue. For

comparison, the sequence of Sso TFS1 was included with the two catalytic acidic residues highlighted in red. **(d)** Phylogenetic distribution of TFS paralogues in the Sulfolobales order. The schematic phylogenetic tree is based on reference [5].

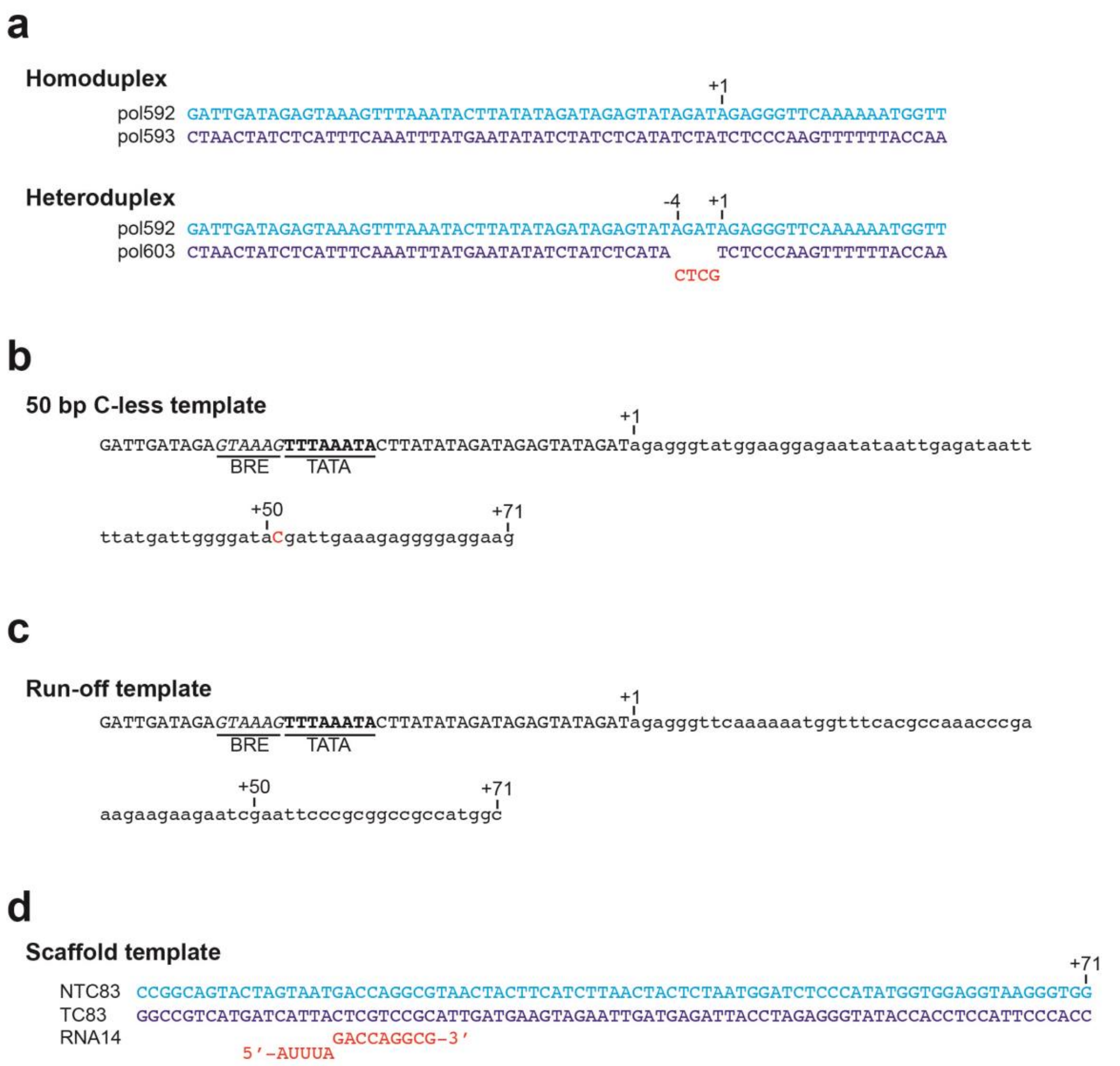


**Supplementary figure 2: DNA templates used for EMSA and transcription assays** (**a**) DNA template sequences used for abortive transcription, potassium permanganate footprinting and PIC EMSA assays. The DNA template strand is in blue, the DNA non-template strand is in cyan. For the heteroduplex template, -4 to -1 mismatch sequence is highlighted in red. (**b**) SSV-T6 promoter 50 nt C-less cassette template used in RNA cleavage and reactivation assays. (**c**) SSV-T6 promoter run-off template used in promoter-directed transcription assays. (**d**) Nucleic acid scaffold sequences used in elongation and TEC EMSA assays. The DNA template strand is in blue, the DNA non-template strand is in cyan, and the RNA is in red.

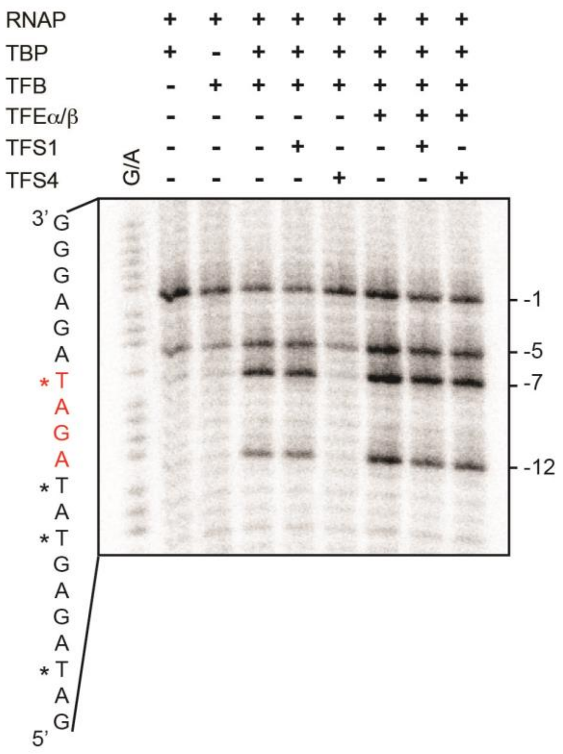


**Supplementary figure 3: Effect of TFS4 on promoter melting.**

The effect of TFS4 on the melting of the SSV1-T6 promoter non-template strand was probed using potassium permanganate foot-printing assays. The promoter template for the assay is identical to the EMSA probes and includes a 4-nt heteroduplex region (register −4 to −1 relative to the TSS, highlighted in red, see also supplementary Fig. S2a). The reactive T residue at position -1 serves as positive control in this assay. The addition of RNAP in the presence of TBP and TFB induces three additional reactive T-residues at positions -5, -7 and -12, which reflect promoter melting in the PIC. The addition of TFEα/β increases their intensity in agreement with the role of TFEα/β during transcription initiation [1]. While the addition of TFS1 has little effect on DNA melting, TFS4 reduces permanganate reactivity to background levels, TFEα/β protects the PIC from TFS4 inhibition, in good agreement with our PIC EMSA. The position of all reactive T residues is indicated with an asterisk. G/A ladder and the corresponding non-template DNA strand sequence are shown on the left.

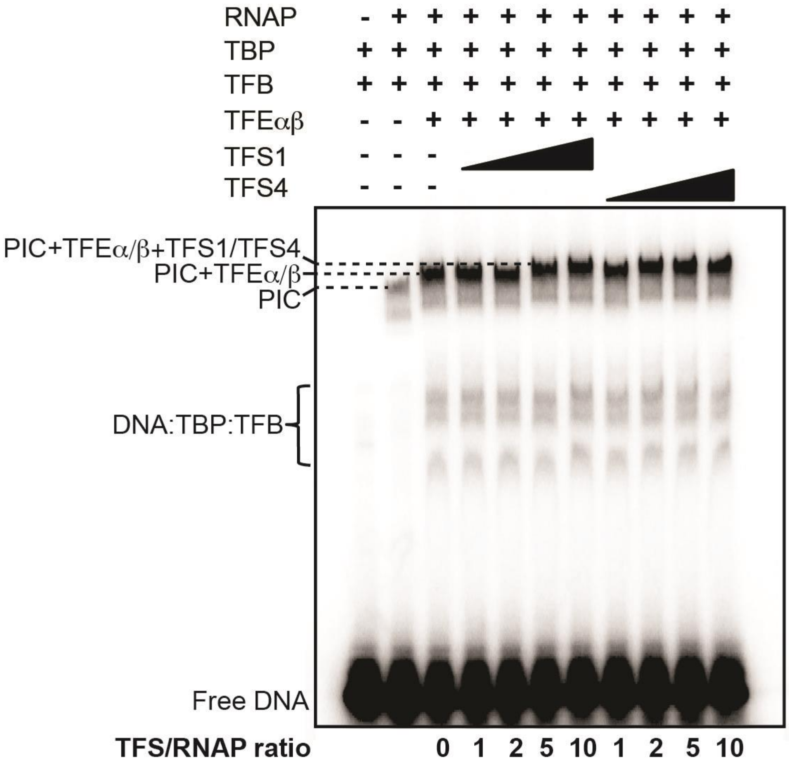


**Supplementary figure 4: TFS1 and TFS4 are incorporated into the TFEα/β-containing complete PIC.**

EMSA assay showing the stepwise assembly of PICs. The addition of TFEα/β to minimal PIC containing DNA-TBP-TFB-RNAP leads to both a slight upshift and an increase in signal intensity of the complete DNA-TBP-TFB-RNAP-TFE PICs. Adding either TFS1 or TFS4 induces a subtle but reproducible additional upshift showing that both can be incorporated into complete PICs. EMSA was carried out in a low salt (150 mM) binding buffer and resolved on a 4-20% Tris-Glycine gel.

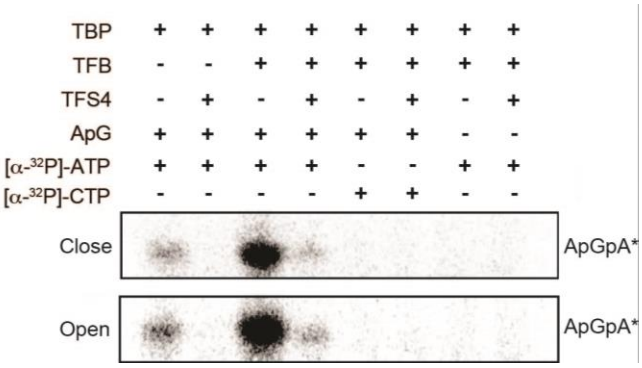


**Supplementary figure 5: Abortive transcription assay controls**

Abortive transcription assays measuring the addition of [α-$^{32}$P]-ATP (correct) or [α-$^{32}$P]-CTP (noncomplementary) to the dinucleotide primer ApG on homoduplex (closed) and heteroduplex (open) SSV1-T6 promoter templates in the presence or absence of 250 nM TFS4.

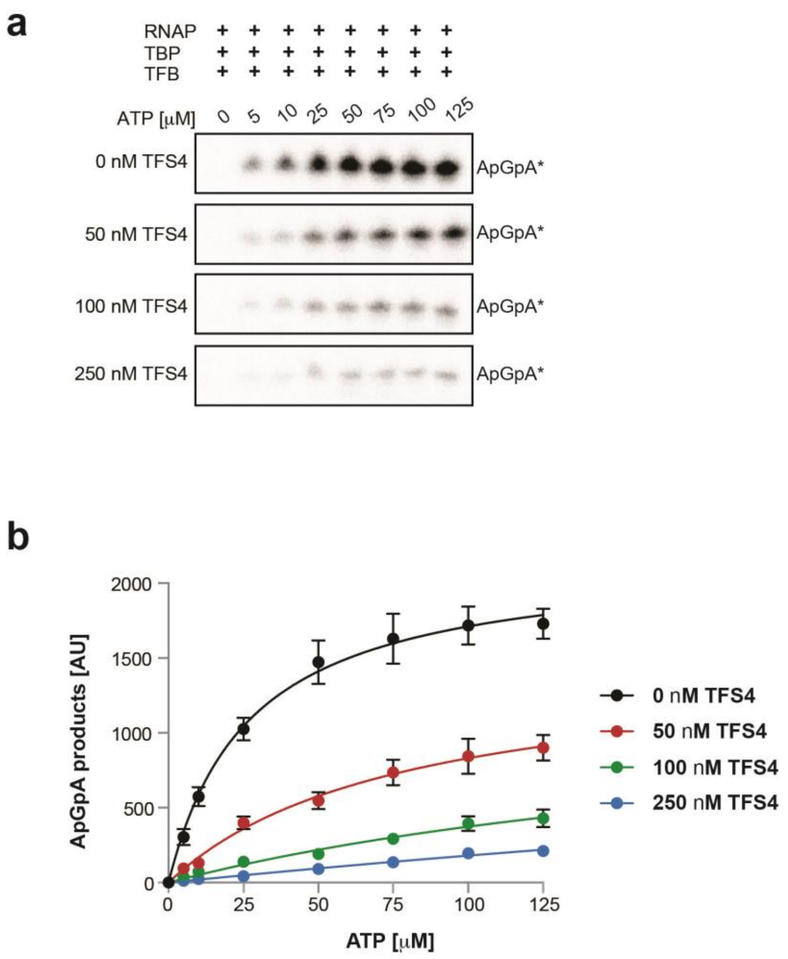


**Supplementary figure 6: Inhibitory effect of TFS4 on abortive initiation at varying substrate NTP concentrations.**

(**a**) We tested the effect of increasing substrate NTP concentration on TFS4 inhibition of transcription in abortive initiation assays at three TFS4 concentrations (50, 100 and 250 nM). (**b**) The 3-nt abortive initiation products were quantified and plotted as a function of ATP concentrations for each TFS4 concentration. Error bars represent standard deviation based on three technical repeats.

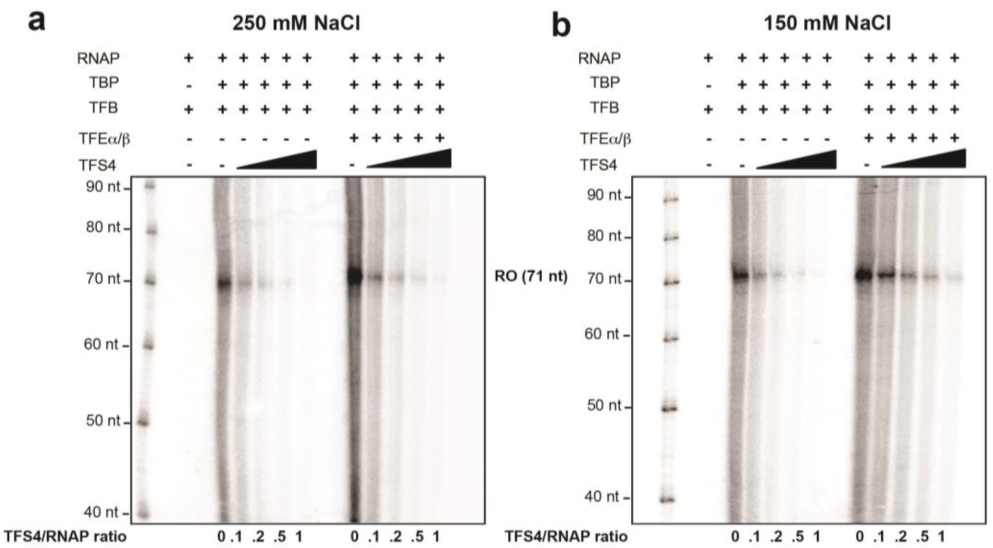


**Supplementary figure 7: TFEα/β interference with TFS4 in promoter-directed transcription assays.**

TFS4 inhibition of transcription in the presence or absence of TFEα/β in promoter-directed transcription assays under (**a**) high- (250 mM) and **(b)** low-salt (150 mM) conditions. In contrast to its interference with PIC formation, TFS4 can inhibit promoter-directed transcription in the presence of TFEα/β, albeit less efficiently than without. This is likely due to the fact that TFS4 interferes with transcription elongation complexes. Reactions were carried out using the same conditions as in Figure 4a.

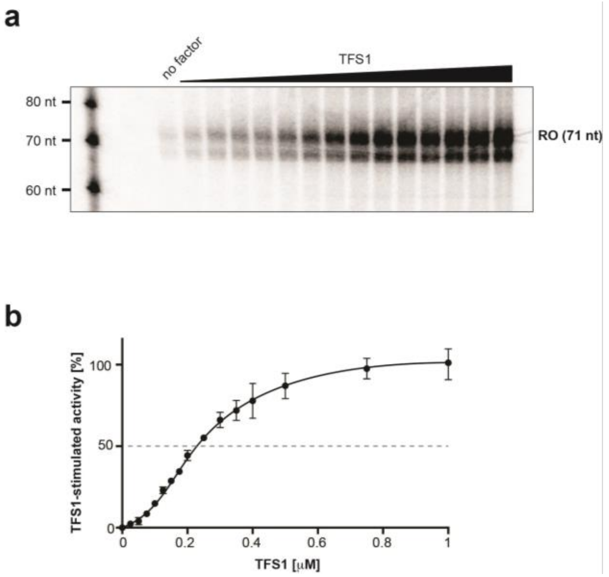


**Supplementary figure 8: TFS1 relieves TFS4 inhibition**

(**a**) The addition of TFS1 counteracts the inhibitory effects of TFS4 in a dose-dependent fashion. In order to compare the apparent relative affinities of RNAP:TFS1 and RNAP:TFS4 complexes, transcription elongation assays were carried out in the presence of 100 nM TFS4 and varying concentrations of TFS1. This concentration of TFS4 is sufficient for complete inhibition of RNAP and therefore assumed to be higher than the apparent $K_m$ (and $K_D$) for RNAP:TFS4 complexes. (**b**) Quantification of transcription elongation assay shown in (a). The relief of inhibition by TFS1 likely reflects the competition of TFS1 and TFS4 for RNAP binding. Under these conditions the $IC_{50}$ of TFS1 is ~230 nM, i.e. the TFS1 concentration required to achieve 50% relief of TFS4 inhibition, where $K_D$ (RNAP:TFS1) /$K_D$ (RNAP:TFS4) = $IC_{50}$ / [TFS4]. The amount of run-off products was quantified using ImageQuant TL Software (GE Healthcare). Error bars represent standard deviation from three technical repeats.

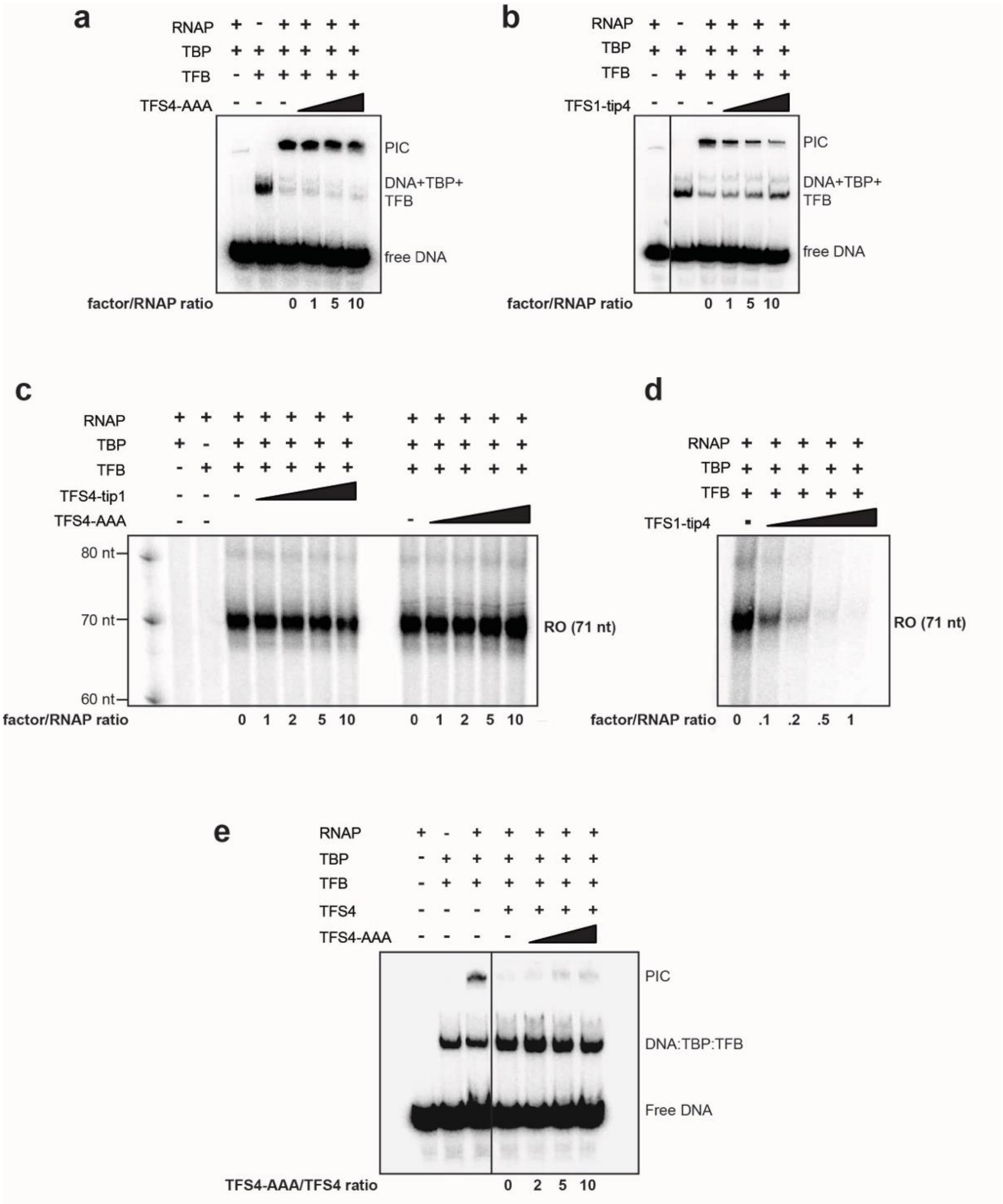


**Supplementary figure 9: Activity of TFS1 and TFS4 variants in EMSA and transcription run-off assays**

(**a**) and (**b**) EMSA with the minimal archaeal PIC complex (SSV1 T6 promoter DNA, TBP, TFB, RNAP) using TFS4-AAA (a) and TFS1-tip4 mutants (b) were carried out as in figure 3. (**c**) and (**d**) Promoter directed transcription assays on the SSV1 T6 promoter template in the presence of TFS4-tip1, TFS4-AAA (c) and TFS1-tip4 (d) mutants were

carried out as in figure 4. (**e**) Increasing amount of TFS4-AAA can only partially rescue the formation of minimal archaeal PIC pre-incubated with TFS4.

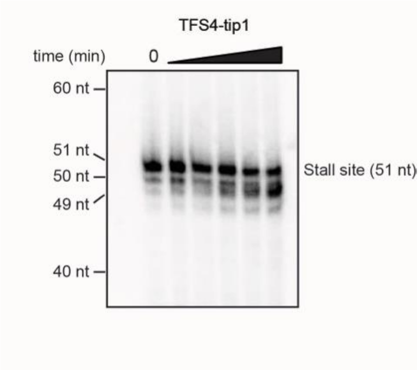


**Supplementary figure 10: The TFS1 tip motif confers transcript cleavage activity to TFS4.**

Transcript cleavage assays were carried out as in figure 2. A chimeric TFS4-tip1 variant encompassing TFS4 and the TFS1 C-ZR tip motif stimulates transcript cleavage in a dose-dependent fashion similar to TFS1. Reactions were carried out using the same conditions as in Figure 2b.

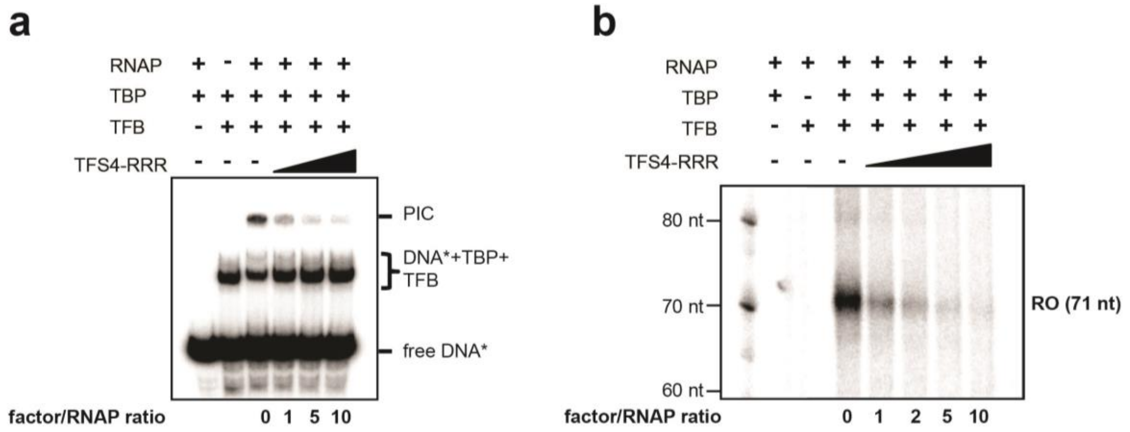


**Supplementary figure 11: Activity of TFS4-RRR in EMSA and transcription run-off assays**

(**a**) EMSA with the minimal archaeal PIC complex (SSV1 T6 promoter DNA, TBP, TFB, RNAP) using the TFS4-RRR mutant was carried out as in Figure 3a. (**b**) Promoter-directed transcription assays using the SSV1-T6 promoter template in the presence of the TFS4-RRR mutant was carried out as in Figure 4a.

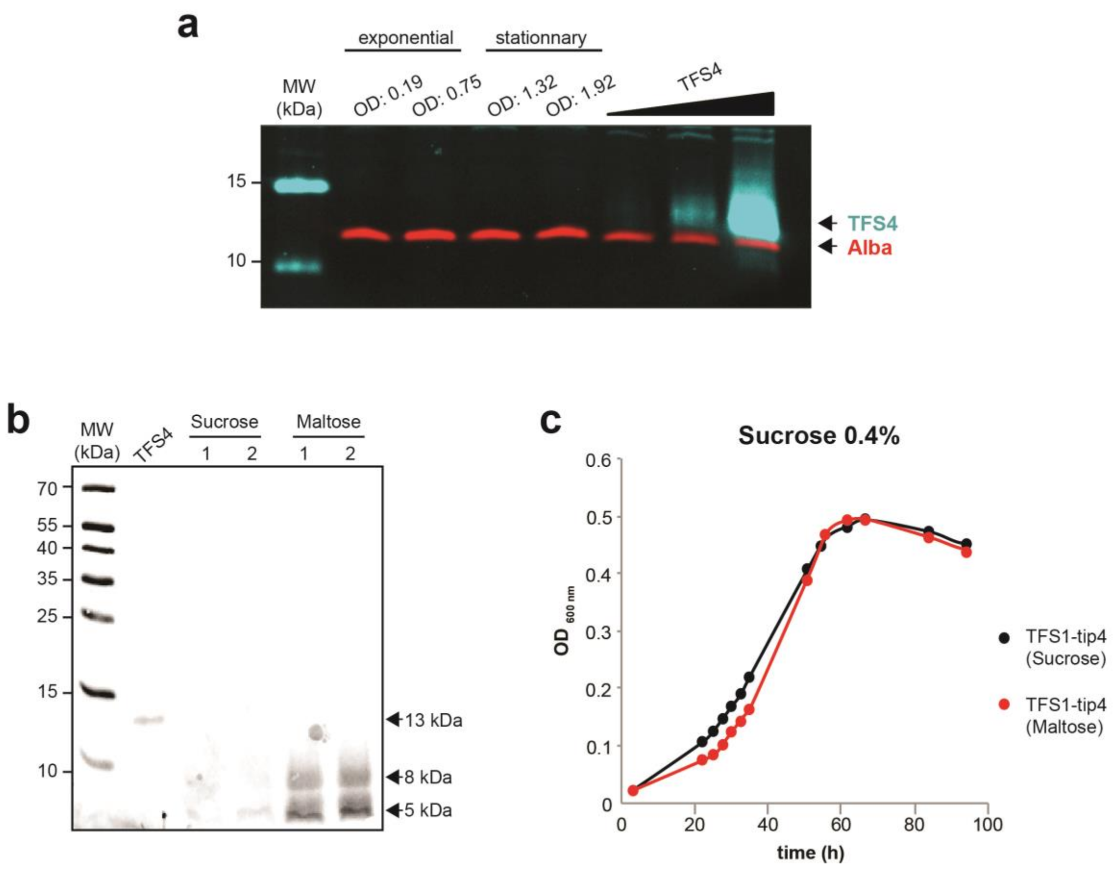


**Supplementary figure 12: TFS4 expression in *Sulfolobus*.**

(**a**) TFS4 is not expressed during either exponential or stationary phase growth in *Sulfolobus solfataricus*. Multiplex Western blot using polyclonal antibodies raised against TFS4 and Alba (loading control) in cell lysates during different growth phases of *Sulfolobus solfataricu*s. 9 µg of total protein was loaded in each lane. The last three lanes contain 0.7, 2.8 and 11.2 ng of recombinant TFS4 spiked into cell lysate in order to serve as positive control. Immunodetection was performed on three biological replicates. (**b**) Immunodetection of vector-driven ectopically expressed Sso TFS4 in *S. acidocaldarius* MW001 in sucrose (non-inducible) and maltose media (inducible). Western blot analysis was performed using polyclonal antibodies raised against Sso TFS4 on two biological replicates. (**c**) Recovery from TFS1-tip4 growth inhibition. The Saci strain harbouring the TFS1-tip4 expression plasmid was cultured under inducing (Maltose) or non-inducing conditions (Sucrose) and both cultures were subsequently

transferred to a non-inducing media (Sucrose). The growth curve is based on at least three biological replicates.

## Supplementary References


1 Blombach, F. *et al.* Archaeal TFEalpha/beta is a hybrid of TFIIE and the RNA polymerase III subcomplex hRPC62/39. *Elife* **4**, e08378, doi:10.7554/eLife.08378 (2015).

2 Rinke, C. *et al.* Insights into the phylogeny and coding potential of microbial dark matter. *Nature* **499**, 431-437, doi:10.1038/nature12352 (2013).

3 Guy, L. & Ettema, T. J. The archaeal 'TACK' superphylum and the origin of eukaryotes. *Trends Microbiol* **19**, 580-587, doi:10.1016/j.tim.2011.09.002 (2011).

4 Zaremba-Niedzwiedzka, K. *et al.* Asgard archaea illuminate the origin of eukaryotic cellular complexity. *Nature* **541**, 353-358, doi:10.1038/nature21031 (2017).

5 Podar, M. *et al.* Insights into archaeal evolution and symbiosis from the genomes of a nanoarchaeon and its inferred crenarchaeal host from Obsidian Pool, Yellowstone National Park. *Biol Direct* **8**, 9, doi:10.1186/1745-6150-8-9 (2013).
